# Effect of Paralysis at the Time of ProSeal Laryngeal Mask Airway Insertion on Pharyngolaryngeal Morbidities. A Randomized Trial

**DOI:** 10.1371/journal.pone.0134130

**Published:** 2015-08-07

**Authors:** Hyo-Seok Na, Young-Tae Jeon, Hyun-Jung Shin, Ah-Young Oh, Hee-Pyoung Park, Jung-Won Hwang

**Affiliations:** 1 Department of Anesthesiology and Pain Medicine, Seoul National University Bundang Hospital, Seongnam, Republic of Korea; 2 Department of Anesthesiology and Pain Medicine, Seoul National University Hospital, Seoul, Republic of Korea; University of Cincinnati College of Medicine, UNITED STATES

## Abstract

Neuromuscular block results in the loss of muscular tone in the upper airway, which might contribute to the increased postoperative airway morbidity followed by ProSeal laryngeal mask airway (PLMA) insertion. We compared the pharyngolaryngeal discomfort exerted by the PLMA according to the neuromuscular block. One hundred sixty patients undergoing surgery for breast disease or inguinal hernia were anesthetized with propofol and remifentanil by target controlled infusion. Rocuronium 0.6 mg/kg (NMBA group, n = 80) or normal saline (No-NMBA group, n = 80) was administered after the loss of consciousness, and one anesthesiologist inserted the PLMA. Postoperative pharyngolaryngeal discomfort was evaluated at postoperative 1 h. Traumatic event was recorded based on the blood trace on the surface of the PLMA cuff. Insertion time, insertion attempt number, sealing pressure, and fiberoptic brochoscopic grades were evaluated. Patients’ characteristics and the PLMA insertion condition (insertion time, successful insertion attempt number, fiberoptic bronchoscopic grade, and sealing pressure) were similar between the two groups. The PLMA can be successfully inserted in non-paralyzed patients with less postoperative pharyngolaryngeal discomfort than when a neuromuscular blocking agent is used (13.8% vs. 30.0%, *P* = 0.021). The incidence of traumatic events is also reduced when no neuromuscular blocking agent is used (16.3% vs. 32.5%, *P* = 0.026). Regardless of whether or not a surgical procedure requires muscular relaxation, there is no need to administer neuromuscular blocking agents solely for the purpose of PLMA insertion.

**Trial Registration:**

ClinicalTrials.gov NCT01035021

## Introduction

The advantages of supraglottic airway devices are that they can reduce pharyngolaryngeal morbidity and make patients recover more smoothly than the endotracheal tube does. Recent studies have verified that numerous pharyngolaryngeal morbidities, including coughing, laryngospasm, hoarseness, and sore throat, occur less frequently when a supraglottic airway is used to secure a patient’s airway rather than endotracheal intubation [1,2]. The supraglottic airway can be inserted by administering an optimal dose of combined propofol and remifentanil without additional neuromuscular blocking agents (NMBA) [3,4]. However, laryngospasm or cough response following supraglottic airway insertion may occur. Furthermore, surgical procedure may require the relaxation of muscle, and ProSeal laryngeal mask airway (PLMA) was inserted in anesthetized, paralyzed patients when it was introduced in the beginning [5]. Thus, NMBA has been still used in various clinical situations, in which general anesthesia is induced for the operation [6–8].

The PLMAis one of a variety of supraglottic airway devices that are widely used during general anesthesia. It differs from the classic laryngeal mask airway (LMA), in that, it is more advantageous for positive pressure ventilation as it creates a more effective seal; it also has a separate drainage passage to enable decompression of gastric distension [9]. The PLMA has been reported to successfully maintain the airway during pelviscopic gynecologic surgery without NMBA [10] as well as during laparoscopic cholecystectomy [11]. Rates of pharyngolaryngeal morbidities associated with the LMA are similar regardless of whether NMBA is used [12]. An earlier comparison of the LMA and PLMA in anesthetized and non-paralyzed patients suggested that the LMA is easier to insert than the PLMA at the first attempt, while postoperative pharyngolaryngeal morbidities are comparable between the two devices [13]. However, there are currently no published date available on pharyngolaryngeal morbidities associated with the use of the PLMA and NMBA.

We hypothesized that neuromuscular block would result in the loss of muscular tone in the upper airway, which might contribute to the increased postoperative airway morbidity followed by PLMA insertion. In the present study, we compared the incidence of pharyngolaryngeal morbidities depending on the use of NMBA, when inserting the PLMA.

## Methods

This randomized double-blind study was approved (Approval Number: B-0905/075-010) by the Institutional Review Board of Seoul National University Bundang Hospital, Republic of Korea on July 2009 and registered in ClinicalTrials.gov (NCT01035021). After getting approval, this study was performed in the Seoul National University Bundang Hospital from July 2009 to April 2010. This study was performed to the patients during the operation under the general anesthesia, and the outcomes were evaluated 1 h after finishing surgery. The written informed consent was obtained from all participants. Patients aged 18–70 years with an American Society of Anesthesiologists physical status of I-II who were scheduled for elective surgeries for breast disease or inguinal hernia under general anesthesia were enrolled. Exclusion criteria were as follows: neuromuscular disease, ongoing pharyngolaryngeal discomfort or disease, a known difficult airway, limited mouth opening, loose teeth, and aspiration risk.

Eligible patients were randomly assigned to one of two groups, the NMBA or No-NMBA group, by opening a sealed envelope before entering the operating room. One anesthesiologist, who did not participate in the anesthetic care, was designated for the preparation of drugs. Rocuronium 0.6 mg kg^-1^ was prepared as a neuromuscular blocking agent, and same volume of isotonic saline was prepared as a placebo drug. The NMBA group received rocuronium before inserting PLMA and No-NMBA group did not received rocuronium before inserting PLMA. However, rocuronium was required for the muscle paralysis during operation in spite of the No-NMBA group. Thus, rocuronium was given to the No-NMBA group after inserting PLMA. For maintaining blinded technique, rocuronium was designated #1 and the isotonic saline #2 in the NMBA group. In the No-NMBA group, isotonic saline was designated #1 and rocuronium #2.

Intravenous midazolam 0.03 mg kg^-1^ was given to each patient to relieve preoperative anxiety. Upon arrival in the operating room, standard monitoring (including electrocardiogram, non-invasive arterial pressure, pulse oximetry, and bispectral index) was established before the induction of anesthesia. Anesthesia was induced with propofol and remifentanil by target-controlled infusion (TCI) using an Orchestra infusion pump system (Fresenius vial, Brezins, France), and the target effect-site concentrations (Ce) were set to 4 μg.ml^-1^ for propofol and 4 ng ml^-1^ for remifentail. Manual ventilation was commenced via a facemask after confirming loss of consciousness and reaching the proper bispectral index value. The prepared #1 drug was then administered: rocuronium for the NMBA group and isotonic saline for the No-NMBA group. After 3 min of mask ventilation, the PLMA was inserted by one experienced anesthesiologist in accordance with the manufacturer’s instructions and without using an introducer tool. A size 4 or 5 PLMA was used for female and male patients, respectively. The cuff was fully deflated and a water-based lubricant applied onto the dorsal side before insertion. After its insertion into the hypopharynx, the cuff of the PLMA was inflated with 15 or 20 ml air depending on its size. Satisfactory sealing of the PLMA was confirmed clinically by square wave capnograph trace and by the lack of any audible leak sound during 8–10 ml kg^-1^ tidal volume ventilation. A maximal inspiratory airway pressure of up to 20 cmH_2_O was permitted during ventilation. After a secure airway was obtained, the ventilator was turned off momentarily, and the sealing pressure was measured by closing the expiratory pop-off valve of the ventilator with 2 l min^-1^ oxygen flow. We determined the sealing pressure at which a leaking sound was heard over the mouth using a stethoscope. During this process, the maximum airway pressure was not allowed to exceed 40 cmH_2_O.

If secure PLMA placement was not established at the first attempt, another attempt was made after its removal. If the second and third attempt failed, the PLMA was inserted by using the 90° rotational technique at fourth attempt [14]. Once proper placement was achieved, the laryngeal area was viewed with a fiberoptic bronchoscope inserted through the airway tube of the PLMA to grade its position. Once these evaluations were complete, the #2 drug was administered to all patients: isotonic saline for the NMBA group and rocuronium for the No-NMBA group.

At the end of the surgery, 30 mg of ketorolac was administered to all patients. In order to reverse the neuromuscular block at the end of surgery, glycopyrrolate 0.01 mg kg^-1^ and neostigmine 0.03 mg kg^-1^ were administered to both groups of patients. During emergence, 100% oxygen was given to all patients and the PLMA was removed once adequate spontaneous ventilation had resumed and the patients regained consciousness. They were questioned regarding pharyngolaryngeal discomfort 1 h after the removal of the PLMA in the post-anesthetic care unit.

The primary outcome was the incidence of complications linked with the use of the PLMA. To evaluate postoperative pharyngolaryngeal discomfort, an observer blinded to the group assignment enquired whether patients experienced any pharyngolaryngeal discomfort 1 h after removal of the PLMA. Pharyngolaryngeal discomforts included a foreign body sensation in the throat, sore throat, dysphagia, hoarseness, and dysphonia. Traumatic events were defined as the presence of visible blood stains on the surface of the PLMA after its removal during emergence.

The secondary outcomes were as follows: insertion time, sealing pressure, number of the PLMA insertion attempts, and the fiberoptic brochoscopic grade for the view.

Insertion time was defined as the brief time from the anesthesiologist picking up the PLMA until the first successful detection of an expiratory CO_2_ wave on the capnography monitor. Sealing pressure was defined as an airway pressure at which no audible leaking sound was heard with a stethoscope around the PLMA when a secure airway was established. The number of PLMA insertion attempts was defined as the number of times a designated anesthesiologist attempt to insert the PLMA until an effective airway was obtained. The laryngeal view was classified into four grades depending on the view from the fiberoptic bronchoscope: grade 1, the vocal cords were not visible; grade 2, the vocal cords and anterior epiglottis were visible; grade 3, the vocal cords and part of the posterior epiglottis were visible; grade 4, only the vocal cord were visible [15].

From our pilot data, we assumed a difference in the incidence of sore throat between the two groups of 21%, and thus at least 71 patients were required in each group for a power of 80% and a risk of type 1 errors of 0.05. Assuming that the dropout rate would be 10%, 80 patients were recruited for each group.

All variables were tested for normal distribution using the Shapiro-Wilk test (results not shown). Data are expressed as the mean ± SD or as a number (%). Statistical analyses were performed using Student’s *t*-test or the Mann-Whitney U test appropriate. The Chi-square test was used to detect the differences in the incidence of pharyngolaryngeal discomfort, number of PLMA insertion attempt, fiberoptic brochoscopic grade, and incidence of traumatic events. Data were analyzed with SPSS software (ver. 21; IBM SPSS), and *P* values < 0.05 were considered statistically significant.

## Results

**Fig 1:**
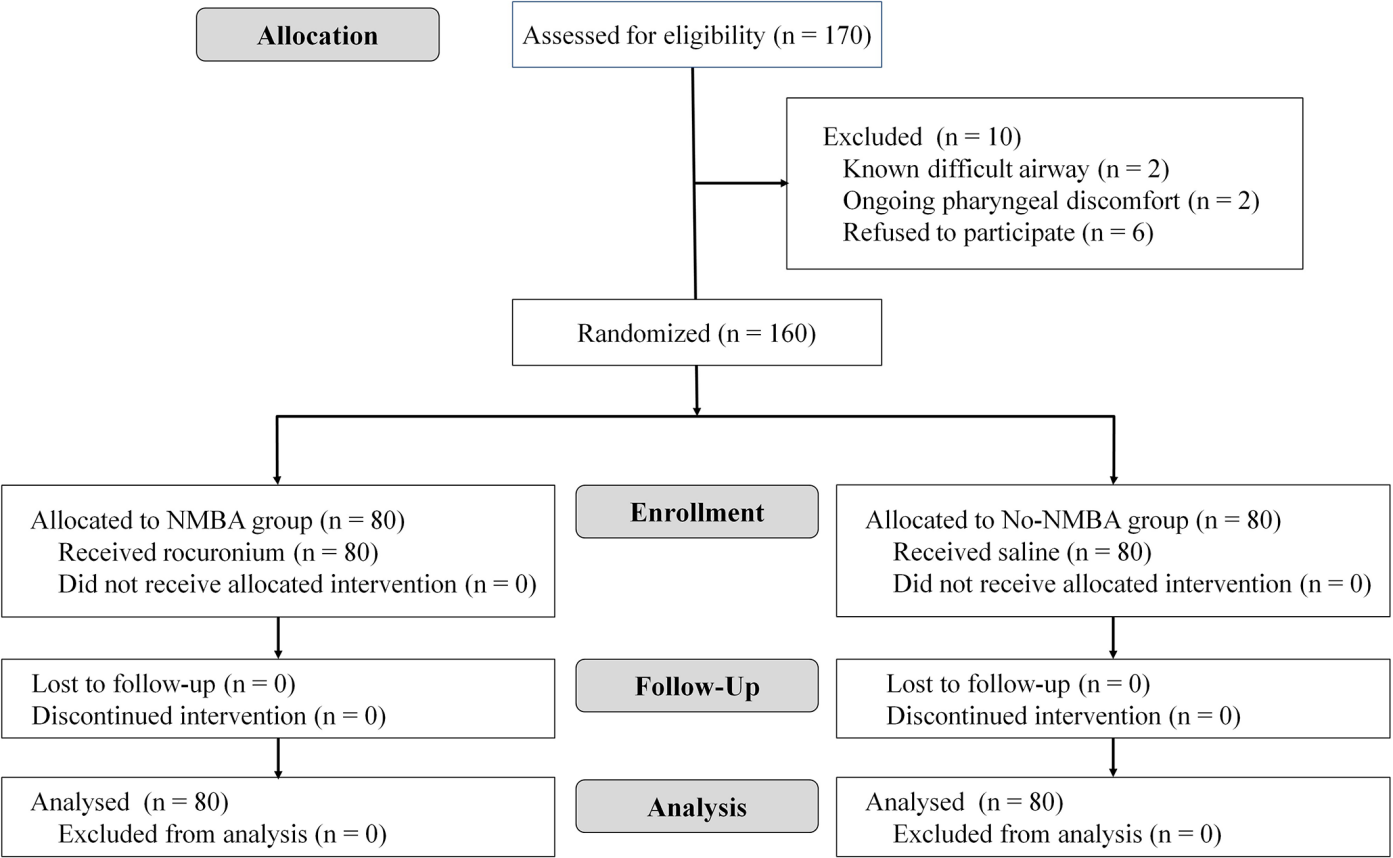
Flow chart of patient enrollment.

A total of 160 patients were enrolled (Fig 1). Patient characteristics and total anesthesia time were similar between the two groups (Table 1).

**Table 1:** Characteristics of patients who received a neuromuscular blocking agent or not at the time of insertion of the PLMA.

	NMBA group (N = 80)	No-NMBA group (N = 80)	*P*-value
**Age (year)**	49.8 ± 12.8	48.9 ± 11.9	0.66
**M/F**	36/44	35/45	1.00
**Height (cm)**	162.2 ± 7.1	160.9 ± 7.5	0.34
**Weight (kg)**	62.2 ± 9.8	59.9 ± 9.2	0.19
**Anesthesia time (min)**	77.0 ± 26.8	79.5 ± 27.9	0.84

Values are expressed as mean ± standard deviation or a number. NMBA, neuromuscular blocking agent.

Postoperative pharyngolaryngeal discomfort occurred less frequently among patients in the No-NMBA group than the NMBA group (13.8% vs. 30.0%, *P* = 0.02). The incidence of traumatic events was also lower in the No-NMBA group that in the NMBA group (16.3% vs. 32.5%, *P* = 0.03) (Table 2). When we analyzed the group of patients in whom the PLMA was inserted at the first attempt, the incidence of postoperative pharyngolaryngeal discomfort was still lower among those in the No-NMBA group than the NMBA group (12.3% vs. 30.2%, *P* = 0.02), as was the incidence of traumatic events (12.3% vs. 27.0%, *P* = 0.05).

**Table 2:** The incidence of pharyngolaryngeal discomfort and traumatic events after removal of the PLMA.

	NMBA group (N = 80)	No-NMBA group (N = 80)	*P*-value
**Pharyngolaryngeal discomfort**	24 (30.0%)	11 (13.8%)	0.02
**Traumatic event**	26 (32.5%)	13 (16.3%)	0.03

Values are expressed as numbers (proportions). NMBA, neuromuscular blocking agent.

The PLMA insertion conditions are shown in Table 3. Rates of successful PLMA insertion were similar between the two groups (*P* = 0.90). It also took a similar amount of time to insert and place the PLMA in the proper position [20.3 (21.4) s vs. 17.9 (19.1) s in NMBA and No-NMBA groups, respectively; *P* = 0.12] In addition, there was no difference in sealing pressures between the NMBA and No-NMBA groups [25.9 (7.4) cmH_2_O vs. 24.7 (7.3) cmH_2_O, respectively; *P* = 0.21]. Finally, there were no significant between-group differences in fiberoptic brochoscopic grade after LMA *ProSeal* insertion (*P* = 0.89).

**Table 3:** Number of PLMA insertion attempts, insertion time, oropharyngeal sealing pressure, and fiberoptic bronchoscope view grades.

	NMBA group (N = 80)	No-NMBA group (N = 80)	*P*-value
**Number of PLMA insertion attempts**			0.90
**1**	63 (78.8%)	65 (81.3%)	
**2**	7 (8.8%)	5 (6.3%)	
**3**	4 (5.0%)	5 (6.3%)	
**4**	6 (7.5%)	5 (6.3%)	
**Insertion time (s)**	20.3 ± 21.4	17.9 ± 19.1	0.12
**Sealing pressure (cmH**_**2**_ **O)**	25.9 ± 7.4	24.7 ± 7.3	0.21
**Grade for fiberoptic bronchoscope view**			0.89
**1, Vocal cords not seen**	19 (23.8%)	17 (21.3%)	
**2, Vocal cords and anterior epiglottis visible**	15 (18.8%)	15 (18.8%)	
**3, Vocal cords and posterior epiglottis visible**	15 (18.8%)	19 (23.7%)	
**4, Only vocal cords visible**	31 (38.8%)	29 (36.3%)	

Values are expressed as mean ± standard deviation or numbers (proportions). NMBA, neuromuscular blocking agent.

## Discussion

We investigated whether there are any differences in the difficulty of PLMA insertion and the incidence of postoperative pharyngolaryngeal discomfort in paralyzed and non-paralyzed patients. We found that neuromuscular block did not affect the difficulty of PLMA insertion, and that the insertion attempt rate and insertion time were comparable between patients who received a NMBA and those who did not. The quality of the secured airway did not differ irrespective of NMBA usage. Moreover, the sealing pressure and the laryngeal view via fiberoptic bronchoscope inserted through the airway tube of PLMA were not show significantly different between the two groups. These findings imply that a NMBA is not necessary for obtaining a better secured airway. Previously, a small dose of mivacurium was reported to improve the insertion of the LMA, but in that study, no opioid was used during the induction period [16]. Lee et al. confirmed that remifentanil improved the insertion of the LMA in non-paralyzed patients [4]. In the present study, propofol and remifentanil were administered in combination by TCI not only for the induction period but also for the maintenance of anesthesia, and this might have been sufficient for easy insertion of the PLMA in both groups of patients.

Various supraglottic airway devices have become popular for use in airway management during general anesthesia, and these do not always require a neuromuscular block for insertion. In a study that was identical our own barring the device used, Hemmerling et al. investigated the insertion profile and postoperative pharyngolaryngeal morbidities of LMA insertion based on the use of NMBA [12]. They reported that neuromuscular block did not influence the incidence and severity of pharyngolaryngeal discomfort or the difficulty of LMA insertion. Another multicentre study compared the insertion conditions and postoperative pharyngolaryngeal morbidities of the LMA and PLMA in anesthetized non-paralyzed patients [13]. They reported that although the PLMA was more difficult to insert than the LMA, it formed a better seal. Postoperative pharyngolaryngeal discomfort was comparable [13]. A further study in anesthetized and paralyzed patients found that the PLMA was more difficult to insert than the LMA unless an introducer tool was used [5]. The PLMA has a larger cuff and a relatively flexible shaft, which may make intraoral positioning and sliding of the cuff into the pharynx more difficult.

The intriguing findings in the present study are the significant differences in the incidence of traumatic events and the rates of pharyngolaryngeal discomfort between patients who did and did not receive a NMBA: PLMA insertion in non-paralyzed patients was less traumatic and resulted in less pharyngolaryngeal discomfort. Although the insertion time and successful insertion rate did not differ, more patients in the NMBA group experienced a traumatic event than in the No-NMBA group, which might have led to greater postoperative pharyngolaryngeal discomfort in the NMBA group.

The skeletal muscle tone of the upper airway should be considered in this context. With increasing depth of anesthesia, the upper airway calibre is decreased at the level of the soft palate and epiglottis in spontaneously breathing children and adults [17,18]. In anesthetized and non-paralyzed but apnoeic patients, the distances of the soft palate, tongue, and epiglottis from the posterior pharyngeal wall are also reduced [19]. Our patients did not breathe spontaneously and positive pressure ventilation was applied after the induction of anesthesia with propofol and remifentanil; thus, it is obvious that the patency of the entire airway was decreased. Sivarajan et al. revealed through radiological examinations that the upper airway structure is altered by a change in head position in anesthetized and paralyzed patients compared with conscious patients [20]. These structures moved forwards or backwards with the flexion and extension, respectively, of the patients head. The oropharynx was narrowed in both head positions after anesthesia and paralysis. This suggests that the PLMA induced more trauma as it passed through this narrow oropharynx area in the NMBA group. In previous studies evaluating postoperative sore throat based on the cuff pressure of the supraglottic airway, low cuff pressure was found to reduce the incidence of postoperative sore throat [21,22]. Keller and Brimacombe reported a positive correlation between the oropharyngeal sealing pressure and directly measured mucosal pressure [23]. We did not measure the mucosal pressure directly; however, the sealing pressure did not differ between the two groups. The extent to which NMBA attenuates pharyngolaryngeal muscular activity in patients deeply sedated by propofol and remifentanil, rendering the pharyngolaryngeal area vulnerable to trauma during PLMA insertion, is not well understood.

An abnormal down-folding of the epiglottis that does not result in the airway obstruction has been commonly reported after the insertion of a supraglottic airway in adult and children [18,24]. Patients in both groups in our study had similar grade distributions for the fiberoptic brochoscopic view, and so the NMBA did not lead to greater posterior deflection of the epiglottis. Further studies are required to determine whether there is a difference in pharyngolaryngeal morbidity based on the position of the epiglottis following supraglottic airway insertion.

The first potential limitation may be that this study was performed in a single center and one expert anesthesiologist of our center inserted the PLMA. These conditions could minimize confounding factors; however, further study including larger cohort and various practitioners would increase reliability. Second, the structural and anatomical change of upper airway or the mucosal pressure according to the use of NMBA was not directly measured in this study. Radiologic evaluation or the measurement of pressure exerted by the PLMA against the pharyngeal mucosa will be required in the next study.

## Conclusions

Postoperative pharyngolaryngeal morbidities were less in the No-NMBA group than the NMBA group. Using an NMBA for the insertion of a PLMA alone for general anesthesia does not facilitate insertion and instead causes greater postoperative pharyngolaryngeal discomfort. Thus, it may be desirable not to administer an NMBA solely for the purpose of inserting a PLMA.

## Supporting Information

S1 File Clinical study protocol in Korean. (DOC)

S2 File Clinical study protocol in English. (DOC)

S3 File CONSORT checklist. (DOC)

S4 File Certificate of Institutional Review Board approval in Korean. (PDF)

S5 File Certificate of Institutional Review Board approval in English. (PDF)

S6 File Sample size calculation. (JPG)
