# Supplementary material for: Effect of Paralysis at the Time of ProSeal Laryngeal Mask Airway Insertion on Pharyngolaryngeal Morbidities. A Randomized Trial
Source: PLoS One. 2015 Aug 7;10(8):e0134130. doi: 10.1371/journal.pone.0134130 (PMC4529079; doi:10.1371/journal.pone.0134130)
Supplement: S1 File — (DOC) [file pone.0134130.s001.doc]

**연구 계획서**

| 마취유도시 근이완제 사용이 ProSeal™ Larygeal Mask Airway 삽입과  수술 후 인후통에 미치는 영향  Effect of neuromuscular blockade on the insertion of ProSeal™ larygeal mask airway and postoperative pharyngolaryngeal discomfort. |
| --- |

제출일시

2009.6.22

분당서울대학교병원

마취통증의학과

나효석

# **연구계획서 요약**

| 연 구 제 목 | 마취유도시 근이완제 사용이 ProSeal™ Larygeal Mask Airway 삽입과 수술 후 인후통에 미치는 영향 |
| --- | --- |
| 연 구 목 적 | 마취 중 ProSeal™ Larygeal Mask Airway를 사용하여 기도 유지를 하는 경우 근이완제의 사용 여부에 따른 삽입 성공률, 삽입 속도, 부작용의 빈도를 비교하고자 한다. |
| 연 구 기 관 | 분당서울대학교병원 마취통증의학과 |
| 연구책임자 | 분당서울대학교병원 마취통증의학과 나효석 |
| 연 구 대 상 | 후두마스크를 삽입하여 마취를 받는 환자 중 연구에 동의한 자. 18세 미만은 제외한다. |
| 연 구 기 간 | 6개월 |
| 연 구 방 법 | - ProSeal™ Larygeal Mask Airway를 삽입하여 마취를 받는 환자 중 연구에 동의한 자를 대상으로 한다.  - 대상을 무작위로 실험군과 대조군으로 배정한다  대조군 (근이완제 사용군)  : 마취 유도 시 근이완제를 사용한 후 후두마스크를 삽입한다..  실험군 (근이완제 비사용군)  : 마취 유도 시 후두마스크 삽입할 때 근이완제를 사용하지 않는다.  - 삽입 성공률, 삽입까지 걸린 시간  - 삽입 전 후 혈압과 심박수를 측정한다.  - 부작용으로서 구강내 출혈 여부, sore throat 여부를 측정한다. |
| 기대효과 및  예상결과 | 후두 마스크의 삽입은 기관 내 삽관에 비해 비숙련자의 경우에도 용이하게 시행할 수 있고 덜 침습적이며 근이완제의 사용 없이도 삽입이 가능한 것으로 알려져 있다. 근이완제를 사용하지 않는 경우라 할지라도 충분한 마취의 깊이가 유지되면 구역반사나 기침, 후두 경련을 예방할 수 있고, 근이완이 되지 않은 상태에서는 인후두부의 구조물들이 해부학적 형태를 유지하게 되므로 후두마스크의 삽입이 더 용이할 것이며 그에 따른 합병증의 발생도 감소할 것이다. |

**목 차**

1. 연구 제목 ------------------------------------------------- 4

2. 실시 기관명 및 주소 ----------------------------------------- 4

3. 연구책임자 및 담당자 ---------------------------------------- 4

3.1 연구책임자

3.2 연구담당자

3.3 공동연구자

4. 연구 배경 및 목적 ------------------------------------------- 5

5. 연구 대상 및 제외 기준 ---------------------------------------- 5

6. 연구 방법 ------------------------------------------------- 6

6.1 연구 방법개요

6.2 목표 피험자의 수 및 산출 근거

6.3 관찰항목, 관찰검사방법 및 임상검사항목

6.4 안전성 및 예상되는 합병증과 해결

7. 예상 연구기간 및 임상시험 일정 --------------------------------- 7

8. 피험자 동의확보 -------------------------------------------- 8

9. 피험자 보상에 관한 규약 (별첨) ---------------------------------- 8

10. 임상기록 양식 (기록지별첨) ------------------------------------ 8

11. 참고문헌 ------------------------------------------------- 8

［ 별첨1 ］ 동의서 -------------------------------------------- 9

［ 별첨2 ］ 피험자 보상에 관한 규약 -------------------------------11

［ 별첨3 ］ 기록지 2 -------------------------------------------12

1. 연구 제목

마취유도시 근이완제 사용이 ProSeal™ larygeal mask airway 삽입과 수술 후 인후통에 미치는 영향

Effect of neuromuscular blockade on the insertion of ProSeal™ larygeal mask airway and postoperative pharyngolaryngeal discomfort.

2. 실시 기관명 및 주소

분당서울대학교병원 마취통증의학과

경기도 성남시 분당구 구미동 300번지

2.1 연구비 지원기관

해당사항 없음

3. 연구 책임자 및 담당자

3.1 연구책임자

분당서울대학교병원 마취통증의학과 전임강사 나효석

3.2 공동연구자

분당서울대학교병원 마취통증의학과 부교수 황정원

분당서울대학교병원 마취통증의학과 조교수 전영태

서울대학교병원 마취통증의학과 조교수 박희평

분당서울대학교병원 산부인과 부교수 김용범

4. 연구 배경 및 목적

전신마취 하 수술 시 기도유지를 하는 방법은 가장 기본적인 기관내 삽관이 있으며, 그 외에 덜 침습적인 여러 방법들이 제시되고 있으며 그 중 하나로 본 연구에서는 사용하게 되는 ProSeal™ LMA (PLMA)도 있다. PLMA는 facial mask가 얼굴을 감싸 구강 및 비강의 기도를 확보하듯이 목 안에서 후두부를 감싸서 기도를 확보하게 해주는 도구로서 PLMA가 제대로 거치되어 있으면 기관내삽관 없이 전신마취가 가능하다.(Fig. 1) 모든 인공 기도 유지기구들은 수술 후 인후통, 출혈 등의 부작용으로 환자에게 수술 후 기간에 수술 부 통증 이외에 또 다른 고통을 줄 수 있다. PLMA 역시 삽입할 당시의 상황에 따라 수술 후 인후부 부작용의 여부가 결정될 수 있다.

Fig. 1

PLMA의 삽입은 기관내 삽관에 비해 비숙련자라 할지라도 용이하게시행할 수 있고 덜 침습적이며 근이완제의 사용 없이도 삽입이 가능한 것으로 알려져 있다. 근이완제를 사용하지 않는 경우라도 충분한 마취의 깊이가 유지되면 구역반사나 기침, 후두 경련을 예방할 수 있고, 근이완이 되지 않은 상태에서는 인후두부의 구조물들이 기존의 해부학적 형태를 유지하게 되므로 후두마스크를 삽입할 때 더 용이할 것이며 그에 따른 인후부 부작용, 즉 통증이나 출혈의 발생도 감소할 것이다.

5. 연구 대상 및 제외 기준

ASA 1,2에 속하고 ProSeal™ LMA (PLMA) 를 삽입하여 마취를 시행받는 환자.

한 군당 91명으로 총 182명

제외기준: 15세 미만의 소아

6. 연구 방법

6.1 연구 방법개요

마취 전 처치는 midazolam 0.04 mg/kg를 수술장 입구에서 정주하고 정맥마취제인 propofol의 지속주입으로 마취를 시행한다.

무작위로 다음의 두 군으로 배정하여 각 군별로 91명씩 모두 182명을 대상으로 한다.

대조군 (근이완제 사용군): 마취 유도 시 rocuronium을 0.6 mg/kg로 정주하여 근이완을 일으킨 후 제조회사 manual에 나와있는 방법을 따라서 오른쪽 검지를 함께 구강에 넣어서 저항이 느껴질 때까지 PLMA를 전진시켜 삽입한다(Fig. 2).

실험군 (근이완제 비사용군): 마취 유도 시 근이완제의 투여 없이 같은 방법으로 PLMA를 삽입한 후 수술을 위해 rocuronium을 0.6 mg/kg를 투여한다.

a.b. c.

Fig. 2.

6.2 목표 피험자의 수 및 산출 근거

피험자의 수는 pilot study 결과 수술 후 인후통의 발생율이 대조군에서 40%, 실험군에서 20%로, 적어도 20%의 발생률이 차이가 나면 이를 인정하고자 한다. 두 군의 수를 같게 하여 80% power와 0.05의 type Ⅰ오차를 가정하고 표본수를 산출하였다.

6.3 관찰항목, 관찰검사방법 및 임상검사항목

연구에 참여하지 않은 간호사가 평가 (1, 4, 5)

(1) 심박수, 평균 혈압 --- PLMA 삽관 전 후 1분에 측정

(2) 삽입시간 --- PLMA를 잡은 시점부터 삽입 후 호흡회로 연결할 때까지 걸린 시간

(3) 삽입 성공률과 시도 횟수 --- 3번까지 삽입을 시도하고 그래도 안되면 실패로 간주한다.

(4) 구강내 출혈 여부 --- 마취가 끝나고 PLMA를 제거할 때 혈액이 묻어있는지 여부

(5) 인후 통증(sore throat) 여부 --- 회복실에서 퇴실시 환자에게 통증여부

6.4 안전성 및 예상되는 합병증과 해결

(1) 저혈압 또는 고혈압의 발생: 삽입으로 인한 혈압 및 심박수의 과도한증가를 막기 위하여 적절한 마취를 시행한다. 그럼에도 불구하고 혈압 상승이 심하면 (수축기 혈압 180 mmHg 이상) 혈압강하제 nicardipine 0.5 mg를 정주한다.

(2) 구강내 출혈 발생: 출혈이 심하면 기도로 흡인이 되지 않도록 처치한다.

(3) 인후 통증: 일반적으로 기도 삽관시보다 발생율이 작지만 심한 통증을 호소할 경우 진통제(ketorolac 30mg ivs)를 투여한다.

(4) 삽입이 어려울 때 저산소증의 발생: 안면마스크를 이용하여 100% 산소로 용수환기하여 저산소증을 치료하고 후두마스크 삽입이 불가능할 경우에는 기관내삽관 등으로 대체한다.

7. 예상 연구기간 및 임상시험 일정

| 데이터 수집 | 보충연구 및 통계분석 |
| --- | --- |
| 6개월 | 4개월 |

현재 연구자가 시행하는 마취 중에 하루에 평균 2 건이 연구대상에 포함된다고 할 때 약 6개월 정도 필요하기에 이를 연구 기간으로 산정하였다.

8. 피험자 동의확보 (동의서별첨)

본 연구는 기관내삽관 없이 PLMA를 사용하여 전신마취를 받는 환자 중에서 PLMA의 삽입 시 일반적으로 발생할 수 있는 모든 부작용과 해결 방안, 본 연구를 위해 근이완제의 주입 시기가 결정될 것임을 설명한 후 이에 동의하는 환자에 한하여 기존의 마취동의서와 별도의 동의서를 받는다.

9. 피험자 보상에 관한 규약 (별첨)

10. 임상기록 양식 (기록지별첨)

11. 참고문헌

1. [Chui PT](http://www.ncbi.nlm.nih.gov/sites/entrez?Db=pubmed&Cmd=Search&Term="Chui PT"%5BAuthor%5D&itool=EntrezSystem2.PEntrez.Pubmed.Pubmed_ResultsPanel.Pubmed_DiscoveryPanel.Pubmed_RVAbstractPlus), [Cheam EW](http://www.ncbi.nlm.nih.gov/sites/entrez?Db=pubmed&Cmd=Search&Term="Cheam EW"%5BAuthor%5D&itool=EntrezSystem2.PEntrez.Pubmed.Pubmed_ResultsPanel.Pubmed_DiscoveryPanel.Pubmed_RVAbstractPlus): The use of low-dose mivacurium to facilitate insertion of the laryngeal mask airway. [Anaesthesia.](javascript:AL_get(this, 'jour', 'Anaesthesia.');) 1998;53:491-5

2. [Hemmerling TM](http://www.ncbi.nlm.nih.gov/sites/entrez?Db=pubmed&Cmd=Search&Term="Hemmerling TM"%5BAuthor%5D&itool=EntrezSystem2.PEntrez.Pubmed.Pubmed_ResultsPanel.Pubmed_DiscoveryPanel.Pubmed_RVAbstractPlus), [Beaulieu P](http://www.ncbi.nlm.nih.gov/sites/entrez?Db=pubmed&Cmd=Search&Term="Beaulieu P"%5BAuthor%5D&itool=EntrezSystem2.PEntrez.Pubmed.Pubmed_ResultsPanel.Pubmed_DiscoveryPanel.Pubmed_RVAbstractPlus), [Jacobi KE](http://www.ncbi.nlm.nih.gov/sites/entrez?Db=pubmed&Cmd=Search&Term="Jacobi KE"%5BAuthor%5D&itool=EntrezSystem2.PEntrez.Pubmed.Pubmed_ResultsPanel.Pubmed_DiscoveryPanel.Pubmed_RVAbstractPlus), [Babin D](http://www.ncbi.nlm.nih.gov/sites/entrez?Db=pubmed&Cmd=Search&Term="Babin D"%5BAuthor%5D&itool=EntrezSystem2.PEntrez.Pubmed.Pubmed_ResultsPanel.Pubmed_DiscoveryPanel.Pubmed_RVAbstractPlus), [Schmidt J](http://www.ncbi.nlm.nih.gov/sites/entrez?Db=pubmed&Cmd=Search&Term="Schmidt J"%5BAuthor%5D&itool=EntrezSystem2.PEntrez.Pubmed.Pubmed_ResultsPanel.Pubmed_DiscoveryPanel.Pubmed_RVAbstractPlus): Neuromuscular blockade does not change the incidence or severity of pharyngolaryngeal discomfort after LMA anesthesia. [Can J Anaesth.](javascript:AL_get(this, 'jour', 'Can J Anaesth.');) 2004;51:728-32

**피험자 설명문 및 동의서**

1. 연구제목: 마취유도시 근이완제 사용이 ProSeal™ Larygeal Mask Airway 삽입과 수술 후 인후통에 미치는 영향

2. 연구 책임자: 마취통증의학과-나효석 (연락처: 031 787 7507)

임상 공동 연구자: 마취통증의학과-황정원, 전영태, 박희평

3. 개요: 이 연구는 자발적으로 참여 의사를 밝히신 분에 한하여 수행 될 것이며, 귀하께서는 본 임상 연구에 참여 의사를 결정하기에 앞서, 다음의 설명을 주의 깊게 읽으시기 바라며, 필요하시면 귀하의 주치의 또는 가족들과 상의하시기 바랍니다. 만일 질문 사항이 있으시면 담당 마취의가 자세하게 설명해 줄 것입니다.

4. 본 연구의 목적 및 내용

본 연구는 후두마스크 삽입 시간의 단축과 부작용 개선을 위한 연구입니다.

전신마취하에 후두마스크 삽입시 근이완제 투여 유무가, 삽입에 소요되는 시간과 수술 후 부작용에 어떤 영향을 미치는지 관찰하게 됩니다.

5. 연구 방법

귀하는 기관내삽관 대신 후두마스크를 이용하여 마취를 시행 받게 됩니다.

본 연구에 참여하시면 무작위로 두 군으로 나누어 한 군은 근이완제 투여 후 후두마스크를 삽입합니다. 다른 한 군은후두마스크 먼저 삽입한 후 수술을 위한 근이완제를 나중에 투여합니다. 이 것 외에 마취종류와 사용되는 마취제의 종류 및 투여방법은 모든 피험자가 똑같습니다. 군 배정은 마취시작시 난수표에 의해 무작위로 배정됩니다. 피험자가 어느 군에 속할지는 미리 알 수 없으며 선택하실 수 없습니다.

6. 본 연구를 통하여 실험군의 피험자가 직접적으로 얻을 수 있는 큰 이득은 없습니다. 단 본 연구를 포함한 일련의 연구들의 결과가 종합된다면 연구에 참여하신 분은 물론 다른 분들 (예를 들면 향후 수술을 받으시는 분들)에게 도움이 될 것입니다.

7. 연구와 관련된 위험요소

본 연구에 사용되는 방법은 그간 본원에서 계속적으로 사용된 방법이며, 마취시 사용되는 후두마스크, 약제 및 감시장치는 연구에 참여하지 않는 환자들과 동일합니다. 두 가지 마취방법을 쳬계적으로 비교하고자 하는 것으로서 기존의 방법과 비교하여 비슷한 부작용을 가지고 있을 것으로 판단됩니다.

두 방법의 공통적인 부작용은 저산소증 및 이에 따른 혈역학적 불안정, 구강내 출혈, 흡입성 폐렴 등이 있습니다. 저산소증의 경우 안면마스크를 이용하여 100% 산소로 용수 환기하면 대부분 금방 회복되며, 삽입이 안될 경우에는 기관내 삽관을 고려 할 수 있습니다. 또한 구강내 출혈의 경우 대부분 저절로 호전이 되며 심각한 경우 지혈제를 묻힌 거즈로 압박함으로써 완화시킬 수 있습니다. 어떤 군에 속하게 되더라도 가능한 모든 부작용은 적절히 감시, 처치될 것입니다.

8. 피해보상

현재 연구에 사용되는 마취도구, 약제들의 종류, 마취 방법은 현재 연구에 참여하지 않는 다른 환자들이 일반적으로 처치 받는 내용과 동일합니다. 그럼에도 불구하고 이들 약제의 사용 및 시술 등 연구 자체와 관련된 문제에 대해 본 연구자들의 책임 주도하에 배상 혹은 보상할 것입니다. 그리고 만약 본 연구와 관련하여 환자분의 정보가 누출되어 이에 의해 피해를 입게 되신다면 이에 대해서는 본 연구자들이 합법적 절차에 따라 배상 혹은 보상하게 될 것입니다.

9. 자발적 참여와 탈락 (연구에 참여하시지 않거나 도중에 그만두실 권리)

환자분은 환자분께서 본 연구에 참여하고자 하실 경우에만 본 연구에 참여하시게 됩니다. 환자분이 원치 않으실 경우 본 연구에 참여하시지 않으셔도 환자분의 진료과정에 어떠한 손해도 없습니다. 환자분은 본인이 원할 때는 언제라도 연구에서 탈락할 권리가 있으며 제공된 자료의 파기를 요청할 수 있습니다. 그 경우 연구 책임자는 즉시 그 자료를 파기할 것입니다. 또한 의사의 판단에 의해 필요할 때는 언제든지 환자분을 연구에서 제외시킬 수 있습니다.

10. 환자의 자료 및 인권보호

귀하의 정보는 본 연구 목적을 제외하고는 본 연구진과 환자 본인 이외의 누구에게도 알려지지 않기 위해 최선의 노력을 기울일 것입니다. 환자분의 정보를 암호화시켜 관리하여 누출되지 않도록 할 것입니다. 다만 본 연구의 절차 및 자료를 확인하기 위하여 본 병원의 임상시험위원회는 귀하에 관한 기록을 접할 수 있습니다. 이러한 행위는 법적으로 귀하의 비밀을 보장하는 한에서 행해질 것입니다. 본 연구는 분당서울대학교병원의 임상시험심사위원회의 심의를 거쳐 실시하는 것이며, 헬싱키 선언과 생명윤리 및 안전에 관한 법률을 준수합니다.

**본인(또는 보호자: 환자가 동의 의사를 표명하기 어려운 상황에 있을 때는 환자에 대해 책임질 수 있는 위치에 있는 보호자가 대신 서명)은 이 설명 및 동의서를 읽고 충분한 설명을 들었으며, 본 연구에 참여할 것을 자발적으로 동의합니다**.

환자와의 관계 :               성명 :                 서명 :

**< 피험자 보상 규약 >**

연구자는 본 임상시험 실시 중 마취에 의한 고혈압 또는 구강내 출혈의 부작용이 나타났을 경우 즉각적으로 적절한 치료를 시행하여 수술중 환자 안전에 최대한 노력을 기울인다.

**1. 이 때 이상 반응 처리는 다음 사항에 적합하여야 한다.**

1) 피험자는 본 시험의 계획서를 충실히 이행하여야 한다.

2) 발생한 고혈압, 구역 및 구토에 대해서는 신속하게 약물(승압제)을 투여하는 등 적절한 치료를 시행하여 혈압을 유지한다.

**2. 다음 경우에는 보상하지 아니한다.**

1) 연구책임자의 후원 하에 시행되지 않았거나 연구자가 제공하지 않은 의약품 및 수술로 인하여 발생한 이상반응의 경우

2) 수술을 위한 환자 자세의 변화로 인해 기도 유지에 관련된 부작용이 발생한 경우

3) 피험자의 부주의로부터 발생된 경우

4) 환자의 기저 질환의 진행 및 악화에 의해 발생한 경우

5) 일반적인 마취의 부작용에 관한 경우

현재 연구에 사용되는 약제들의 종류, 투약방법, 사용량은 현재 연구에 참여하지 않는 다른 환자들이 일반적으로 처치 받는 내용과 동일하다. 통상적 진료와 무관하게 본 연구와 관련된 문제가 발생시 이에 대한 처리는 본 연구자들이 합법적 절차에 따라 배상 혹은 보상하게 될 것이다.

앞에서 언급한 여러 제반 내용을 참고하여, 피험자가 본 시험에 의해 불이익을 받지 않도록 주의할 것을 서약합니다.

날짜: 2009. 5. 21.

연구 책임자 : 나 효 석

|  |
| --- |

**임상 기록 양식**

| Group / No |  | Sex/Age |  |
| --- | --- | --- | --- |
| Ht/Wt |  | Size of LMA |  |
| Anesthetic time |  |  |  |
| Insertion time (grasp to ventilation) |  | No of Trial | 1 / 2 / 3 / 4 |
| Bronchoscopic grade | VC / post / ant / no-VC | Sealing Pr. |  |
| Traumatic event | | | |
| Blood on LMA | Y / N |  |  |
| Pharyngolaryngeal discomforts | | | |
| Sore throat | Y / N | Hoarseness | Y / N |
| Foreign body sensation | Y / N | Dysphagia | Y / N |
| Dysphonia | Y / N |  |  |
|  | |  | |
