# Supplementary material for: Effect of Paralysis at the Time of ProSeal Laryngeal Mask Airway Insertion on Pharyngolaryngeal Morbidities. A Randomized Trial
Source: PLoS One. 2015 Aug 7;10(8):e0134130. doi: 10.1371/journal.pone.0134130 (PMC4529079; doi:10.1371/journal.pone.0134130)
Supplement: S2 File — (DOC) [file pone.0134130.s002.doc]

**Clinical Research Proposal**

| Effect of neuromuscular blockade on the insertion of ProSeal™ larygeal mask airway and postoperative pharyngolaryngeal discomfort. |
| --- |

2009.6.22.

Seoul National University Bundang Hospital

Anesthesia and Pain Medicine

Hyo-Seok Na

**Organization and location**

Department of Anesthesia and Pain Medicine

Seoul National University Bundang Hospital

Seongnam, Gyeonggi, South Korea

**Background**

- The basic method to secure airway during general anesthesia is intubation.
- Inserting ProSeal™ LMA (PLMA) is one of the non-invasive methods.
- PLMA is designed to conform to the contours of the hypopharynx and can be used with spontaneously breathing patients.

(Fig. 1.)

- PLMA is also designed for use with positive pressure ventilation, with or without neuromuscular block.
- If no neuromuscular blocking agent was used, pharyngolaryngeal structure might keep in its anatomic shape, therefore, pharyngolaryngeal discomfort would occur less.

**Inclusion and Exclusion criteria**

*Inclusion criteria*

- Patients undergoing operation under general anesthesia using PLMA

- American Society of Anesthesiologist physical status I or II

*Exclusion criteria*

- Patients under 15 or less

**Brief design**

*Premedication*

- Midazolam 0.03 mg/kg at reception area of operating room

*Anesthesia*

- Propofol and remifentanil by target-controlled infusion
- Target effect-site concentrations: 4 μg.ml^-1^ for propofol and 4 ng ml^-1^ for remifentail.

*Group arrangement and treatment*

- Sealed envelope method for randomization
- Rocuronium 0.6 mg kg^-1^ was prepared as a neuromuscular blocking agent with the corresponding volume of isotonic saline.
- For the NMBA group, rocuronium was designated #1 and the isotonic saline #2.
- For the No-NMBA group, isotonic saline was designated #1 and rocuronium #2.

*PLMA insertion technique*

a.b. c.

- A size 4 or 5 LMA *ProSeal*™ for female and male patients

- The cuff of the LMA *ProSeal*™ was inflated with 15 or 20 ml air.

**Enrolled patient number**

Detecting difference in the incidence of pharyngolaryngeal discomfort between the two groups of 20%

A power of 80% and a risk of type 1 errors of 0.05.

**Outcome measures**

*Insertion time*

- The brief time from picking up the PLMA until the detection of an expiratory CO_2_ wave on the capnography monitor

*Number of PLMA insertion attempts*

*Traumatic events*

- The visible blood stains on the surface of the PLAM after its removal.

*Pharyngolaryngeal discomforts*

- Foreign body sensation in the throat, sore throat, dysphagia, hoarseness, and dysphonia

**Safety, expected complication, and management**

*Hypotension, hypertension, bradycardia, or tachycardia*

- Appropriate depth of anesthesia,
- Systolic pressure > 180 mmHg: nicardipine 0.5 mg

*Intraoral bleeding*

- Suction for preventing aspiration

*Pharyngolaryngeal pain*

- Ketorolac 30 mg

*Hypoxia caused by failed PLMA insertion*

- Manual ventilation with 100% oxygen and intubation

**Study schedule**

Collection of data: 4 months after approval of IRB

Statistics and supplementary study: 2 months

|  |
| --- |

**Case Report Form**

| Group / No |  | Sex/Age |  |
| --- | --- | --- | --- |
| Ht/Wt |  | Size of LMA |  |
| Anesthetic time |  |  |  |
| Insertion time (grasp to ventilation) |  | No of Trial | 1 / 2 / 3 / 4 |
| Bronchoscopic grade | VC / post / ant / no-VC | Sealing Pr. |  |
| Traumatic event | | | |
| Blood on LMA | Y / N |  |  |
| Pharyngolaryngeal discomforts | | | |
| Sore throat | Y / N | Hoarseness | Y / N |
| Foreign body sensation | Y / N | Dysphagia | Y / N |
| Dysphonia | Y / N |  |  |
|  | |  | |
