# Supplementary material for: Effect of Paralysis at the Time of ProSeal Laryngeal Mask Airway Insertion on Pharyngolaryngeal Morbidities. A Randomized Trial
Source: PLoS One. 2015 Aug 7;10(8):e0134130. doi: 10.1371/journal.pone.0134130 (PMC4529079; doi:10.1371/journal.pone.0134130)
Supplement: S4 File — (PDF) [file pone.0134130.s004.pdf]

분당서울대학교병원 생명윤리심의위원회

Tel : 82-31-787-1376~1378
Fax : 82-31-787-4025 경기도 성남시 분당구 구미로 173번길 82 (우) 463-707

본 위원회에서 승인된 모든 연구자들은 다음의 사항을 준수하셔야 합니다.

1. 연구계획서 및 변경계획서의 승인 이전에 연구대상자의 해당 임상연구의 참여 금지됩니다.

2. 승인 받은 계획서에 따라 연구를 수행하여야 합니다. 변경계획서에 대한 승인 이전에 원 임상연구 계획서와 다른 임상연구의 실시는 금지됩니다.

3. IRB 승인 받은 동의서를 사용하여야 합니다.

4. 연구대상자에게 강제 혹은 부당한 영향이 없는 상태에서 충분한 설명에 근거하여 동의과정을 수행할 것이며, 잠재적인 연구대상자에게 연구에의 참여여부를 고려할 수 있도록 충분한 기회를 제공하여야 합니다.

5. 연구진행에 있어 연구대상자를 보호하기 위해 불가피한 경우를 제외하고 연구의 어떠한 변경이든 위원회의 사전승인을 받고 수행하여야 합니다. 연구대상자들의 보호를 위해 취해진 어떠한 응급상황에서의 변경도 즉각 위원회에 보고하여야 합니다.

6. 연구대상자에게 발생한 즉각적 위험 요소의 제거가 필요하여 원 계획서와 다르게 연구를 실시해야하는 경우, 연구대상자에게 발생하는 위험요소를 증가 시키거나 연구의 실시에 중대한 영향을 미칠 수 있는 변경사항, 예상하지 못한 중대한 이상약물/의료기기 반응에 관한 사항, 연구대상자의 안전성이나 임상 연구의 실시에 부정적인 영향 을 미칠 수 있는 새로운 정보에 관한 사항은 위원회에 신속히 보고하여야 합니다.

7. 위원회의 승인을 받은 연구대상자 모집 광고문을 사용해야 합니다.

8. 위원회의 승인은 1년을 초과할 수 없습니다. 1년 이상 연구를 지속하고자 하는 경우에는 반드시 연차지속보고를 하여야 하며, 위원회에서 요구한 중간보고주기에 따라 연구 진행과 관련한 보고서를 제출하여야 합니다.

9. 심의결과가 승인이 아닌 경우에는 답변서를 제출하여야 하며, 심의일로부터 6개월 이내에 이루어져야합니다.

10. 위원회가 연구를 반려하는 경우 이의신청을 할 수 있으며, 같은 사항에 대하여 2번 연속으로 이의 신청은 할 수 없습니다.

11. 연구종료 시에는 종료 및 결과보고서를 작성하여 제출해야 합니다.

12. 생명윤리 및 안전에 관한 법률, 약사법/의료기기법, 헬싱키 선언 및 ICH-GCP 가이드라인 등 국내외 관련 법규를 준수하여야 합니다.

13. 헬싱키선언에 따라 모든 임상시험은 첫 연구대상자를 모집하기 전 공개적으로 접근이 가능한 데이터베이스 (primary registry)에 연구에 대하여 공개하여야 하며, 예를 들어 http://register.clinicaltrials.gov 를 이용 하실 수 있습니다. 상세한 내용은 IRB 홈페이지를 참고하십시오.

14. 승인 받은 연구에 대하여 기관의 내부 점검 및 외부의 실태조사를 받을 수 있습니다. 기관의 내부 점검자, 외부의 모니터요원 및 점검자, 규제기관의 실태조사자 등이 연구 관련 문서(전자문서 포함)에 대한 열람을 요청하는 경우 연구담당자는 이에 적극 협조해야 합니다.

분당서울대학교병원 생명윤리심의위원회

Tel : 82-31-787-1376~1378
Fax : 82-31-787-4025 경기도 성남시 분당구 구미로 173번길 82 (우) 463-707

심의결과통보서

IRB No.	B-0905/075-010		제출경로	분당서울대학교병원	
연구 과제명	(국문)	마취유도시 근이완제 사용이 ProSeal™ Larygeal Mask Airway 삽입과 수술 후 인후통에 미치는 영향			
	(영문)				
	Protocol No.		Version No.		
연구자		성명	소속	직위	전공분야
	책임연구자	나효석	마취통증의학과	교수(촉탁교수이상)	
	의뢰기관				
생명윤리 및 안전에 관한 법률에 따른 분류	☐ 인간대상연구 ☐ 인체유래물연구 ☐ 배아줄기세포주이용연구 ☐ 배아연구<br>☐ 체세포복제배아연구 ☐ 단성생식배아연구 ☐ 배아생성의료기관 ☐ 인체유래물은행				
연구 종류	임상시험 외 연구	☐ 증례보고 ☐ 생태학적 연구 ☐ 단면조사 연구<br>☐ 조사,설문,인터뷰 연구 ☐ 환자군 연구(case series) ☐ 환자-대조군연구<br>☐ 인체유래물 저장소 연구 ☐ 등록(레지스트리)연구<br>☐ 시판후사용성적조사(PMS) ☐ 전향적 코호트 연구 ☐ 후향적 코호트 연구<br>☐ 기타 ( )			
	임상시험	연구대상	☐ 의약품 ☐ 생물학적제재 ☐ 화장품 ☐ 건강기능식품<br>☐ 의료기기(분류번호(등급):) ▣ 기타 (약물주입시기에 따른 시술의 시점변화)		
			일반명		상품명
		Phase	☐ 제 1 상 ☐ 제 1/2 상 ☐ 제 2 상 ☐ 제 2/3 상 ☐ 제 3 상 ☐ 제 4 상<br>☐ 생물학적동등성 ☐ PMS 연구 ☐ Phase 분류 없음 ☐ 기타 ( )		
		식약처 승인 대상 여부	☐ 식약처 승인 대상 ☐ 승인 제외 대상<br>* 식약처 승인 절차 진행 중인 경우 추 후 식약처 승인서 제출 바랍니다.		
		임상시험 목적	☐ 학술용 ☐ 국내(KFDA)허가용<br>☐ 해외 허가용 (국가명 :)		
연구계획서승인일	2009년 07월 01일		(정기보고주기 : 1년)		
승인유효 만료일	~ 2010년 06월 30일 까지				
심의종류	☐ 정규심의 ▣ 신속심의 ☐ 긴급심의		심의대상	검토의견에대한답변서	
접수일자			심의일자	2009년 07월 01일	
심의결과통보일	2009년 07월 08일				
심의목록	■ 시정승인결정에 대한 답변서<br>■ 연구계획서<br>■ 피험자 설명문 및 동의서<br>■ 피험자보상규약				
심의결과	승인				
심의의견	검토완료				

본 통보서에 기재된 사항은 분당서울대학교병원 생명윤리심의위원회의 기록된 내용과 일치함을 증명합니다.
본 생명윤리심의위원회는 생명윤리 및 안전에 관한 법률, 약사법, 의료기기법 및 ICH-GCP등 관련 법규를 준수합니다.
본 연구와 이해관계(Conflict of Interest)가 있는 위원이 있을 경우 연구의 심의에서 배제하였습니다.

분당서울대학교병원 생명윤리심의위원회

Tel : 82-31-787-1376~1378
Fax : 82-31-787-4025
경기도 성남시 분당구 구미로 173번길 82 (우) 463-707

생 명 윤 리 심 의 위 원 회 위 원 장

본 통보서에 기재된 사항은 분당서울대학교병원 생명윤리심의위원회의 기록된 내용과 일치함을 증명합니다.
본 생명윤리심의위원회는 생명윤리 및 안전에 관한 법률, 약사법, 의료기기법 및 ICH-GCP등 관련 법규를 준수합니다.
본 연구와 이해관계(Conflict of Interest)가 있는 위원이 있을 경우 연구의 심의에서 배제하였습니다.
