# Supplementary figures and images for: Effect of Paralysis at the Time of ProSeal Laryngeal Mask Airway Insertion on Pharyngolaryngeal Morbidities. A Randomized Trial
Source: PLoS One. 2015 Aug 7;10(8):e0134130. doi: 10.1371/journal.pone.0134130 (PMC4529079; doi:10.1371/journal.pone.0134130)

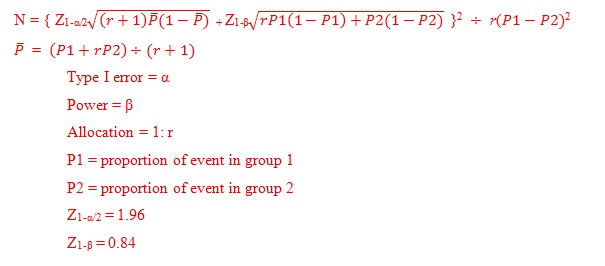

Supplement: S6 File — (JPG) [file pone.0134130.s006.jpg]
